# EMILIN2 Regulates Platelet Activation, Thrombus Formation, and Clot Retraction

**DOI:** 10.1371/journal.pone.0115284

**Published:** 2015-02-06

**Authors:** Menggui Huang, Devaraja Sannaningaiah, Nan Zhao, Yanqing Gong, Jessica Grondolsky, Jane Hoover-Plow

**Affiliations:** Department of Molecular Cardiology, Cleveland Clinic Lerner Research Institute, Cleveland, Ohio, United States of America; University of Munich, GERMANY

## Abstract

Thrombosis, like other cardiovascular diseases, has a strong genetic component, with largely unknown determinants. EMILIN2, Elastin Microfibril Interface Located Protein2, was identified as a candidate gene for thrombosis in mouse and human quantitative trait loci studies. EMILIN2 is expressed during cardiovascular development, on cardiac stem cells, and in heart tissue in animal models of heart disease. In humans, the EMILIN2 gene is located on the short arm of Chromosome 18, and patients with partial and complete deletion of this chromosome region have cardiac malformations. To understand the basis for the thrombotic risk associated with EMILIN2, EMILIN2 deficient mice were generated. The findings of this study indicate that EMILIN2 influences platelet aggregation induced by adenosine diphosphate, collagen, and thrombin with both EMILIN2-deficient platelets and EMILIN2-deficient plasma contributing to the impaired aggregation response. Purified EMILIN2 added to platelets accelerated platelet aggregation and reduced clotting time when added to EMILIN2-deficient mouse and human plasma. Carotid occlusion time was 2-fold longer in mice with platelet-specific EMILIN2 deficiency, but stability of the clot was reduced in mice with both global EMILIN2 deficiency and with platelet-specific EMILIN2 deficiency. *In vitro* clot retraction was markedly decreased in EMILIN2 deficient mice, indicating that platelet outside-in signaling was dependent on EMILIN2. EMILIN1 deficient mice and EMILIN2:EMILIN1 double deficient mice had suppressed platelet aggregation and delayed clot retraction similar to EMILIN2 mice, but EMILIN2 and EMILIN1 had opposing affects on clot retraction, suggesting that EMILIN1 may attenuate the effects of EMILIN2 on platelet aggregation and thrombosis. In conclusion, these studies identify multiple influences of EMILIN2 in pathophysiology and suggest that its role as a prothrombotic risk factor may arise from its effects on platelet aggregation and platelet mediated clot retraction.

## Introduction

EMILIN2 (E2) is a 116 kD extracellular matrix glycoprotein [1,2] with five protein domains: C-terminal C1q domain, proline-rich domain, collagenous domain, coiled-coil domain, and N-terminal cysteine-rich domain (EMI domain). E2 was first identified as a binding partner to EMILIN1 (E1) and both are elastin microfibril interface proteins [1]. Microfibrils consisting of a fibrillin scaffold, elastin, and other proteins, including the EMILINs, are assembled outside the cell to form elastic fibers that impart the elasticity to tissue and vessels. The large number of mutations found in the microfibrillar proteins that cause a broad range of serious defects highlight the importance of the microfibrillar proteins in pathophysiology [3,4]. Elastin, fibulins, fibrillar proteins, and thrombospondins are all microfibril constituents and are all required for recovery from injury and prevention of vascular disease [5]. In addition, other elastin/microfibril proteins, namely fibulin-1 [6,7], multimerin-1 (MMNR1) [8], and microfibril-associated glycoprotein 1(MAGP1) [9], function in hemostasis and thrombosis.

Our interest in EMILIN2 (E2) arose from a screen for genes associated with thrombosis. In our study, a panel of 21 chromosome substitution strains were screened with a tail-bleeding/rebleeding assay, a reporter assay for hemostasis and thrombosis [10]. We identified a quantitative trait locus (QTL) on mouse chromosome 17 as a modifier of thrombosis [11,12]. E2 was identified as a candidate gene within the locus. Previously, a QTL in humans had been identified in the homologous region on human Chromosome 18, and E2 was among the genes in this region [13]. Our initial observation to implicate E2 in platelet function came from our study in which an E2 antibody suppressed platelet aggregation induced by ADP compared to the control IgG, and E2 immunostaining of the thrombus from the FeCl_3_ carotid injury model displayed a expression pattern similar to P-selectin from platelets [14]. To examine the role of E2 in platelet function, E2 conditional knockout mice were developed.

The results of this study indicate that E2 is necessary for platelet aggregation, clot retraction, and thrombus formation *in vivo*. Since E2 and E1 are reported to colocalize in the ECM and to form a heterodimer [15], E1 deficiency and double deficient (E2^-/-^:E1^-/-^) mice also were developed and were found to have suppressed platelet aggregation, suggesting that both E2 and E1 influence platelet responses. E1 expression was reduced in E2^-/-^ mice, but E2 expression was increased in E1D mice. E2 and E1 had the opposite effects on clot retraction with E2 deficiency markedly suppressing clot retraction and E1 markedly increasing clot retraction. Thus, the two EMILIN proteins, E2 and E1, may work in concert to regulate platelet aggregation and clot retraction.

## Materials and Methods

### Mice

To generate the E2 deficient mice in a C57BL/6 (B6) background two lox-P sites were inserted flanking exon 3 in the mouse genome (Taconic). E2^flox/flox^ mice were crossed with the CMV-Cre recombinant mice to generate the global E2 deficient (E2^-/-^) mice that are compared to wild-type mice with CMV-Cre(WTc). The Pf4-Cre recombinant mice were crossed with E2^flox/flox^ to generate the E2 platelet deficient mice (E2p^-/-^) that are compared to wild-type mice with Pf4-cre (WTp). Genopyting was performed by using primers LoxPF: GAGTCGTGAGTGCCCTTGCCA; LoxPR: GGAGACAACCCATCCCCACAATAT; CreR: CCTGTGCTTTACAAACATACATTCATTCACTAATG; CMV-Cre-F: GCGGTCTGGCAGTAAAAACTATC; CMV-Cre-R: GTGAAACAGCATTGCTGTCACTT; Pf4-Cre-F: CCCATACAGCACACCTTTTG; Pf4-Cre-R: TGCACAGTCAGCAGGTT. Professor Giorgio Bressan, University Padua, Italy, kindly provided E1 deficient (E1D) mice in a B6 background, and B6 mice were used as the control (WT) for E1D mice. E2^-/-^ and E1^-/-^ mice were bred to obtain heterozygous mice, genotyped and bred to generate the E2^-/-^:E1^-/-^ homozygous double deficient mice. All mice were maintained on C57BL/6 background and utilized at 8–10 wk of age. Mice were bred, housed in sterilized isolator cages, maintained on a 14-hour light/10-hour dark cycle, and provided with sterilized food and water at the Biological Resource Unit of the Lerner Research Institute of the Cleveland Clinic. All animal experiments were performed in accordance with protocols approved by the Cleveland Clinic Institutional Animal Research Committee.

### qRT-PCR

Total RNA was purified from the mouse tissues using Qiagen RNeasy Mini Kit (Qiagen), digested with TURBO DNase (Ambion) and reverse transcripted into cDNA using SuperScript III First-Strand Synthesis System for RT-PCR (Invitrogen, Carlsbad, CA) according to the manufacturer’s instructions. Real-time PCR was performed using a BioRad iCycler iQ (BioRad, Hercules, CA). Each amplification reaction contained 20 ng of cDNA, 300 nM of each primer, 25 μL of 2X power SYBR Green Master Mix (Applied Biosystems, Warrington, UK), and 0.5μL of UNG (Applied Biosystems) added to prevent carryovers. Samples were normalized to GAPDH expression. The comparative cycle threshold method was used to analyze the data. RNA was isolated from 5–6 mice for each group and analyzed in triplicates. Primers for *Emilin2* qPCR were: EMILIN2-F: GGCCCCCATCAGCTCGACCAAAAA; EMILIN2-R: GTGCAGGGGACCGAGCCAGGAGT.

### Platelet Aggregation

Blood was collected into sodium citrate from the vena cava of anesthetized mice. The platelet rich plasma (PRP) was collected by centrifugation at 100 x g for 12 min, and platelet were removed by centrifugation at 1000 x g for 12 min to obtain platelet poor plasma (PPP). Platelets were counted using a Cellometer Auto M10 (Nexcelom Bioscience, Lawrence, MA) and adjusted with PPP to 0.5–2.0 ×1 0^8^/mL for aggregation studies. To isolate the platelets, the PRP was centrifuged for 15 minutes, and the resuspended platelets were applied onto a CL2B column and eluted with Tyrode’s buffer containing 0.35% BSA and 0.1% glucose, and purified by gel filtration on a Sepharose 2B column [16]. Platelet aggregation was measured in an aggregometer (Chrono-log Corporation), initiated by the addition of agonist, native collagen fibrils, type I collagen (Chrono-Par Aggregation Reagents, No. 385), ADP, or thrombin, and followed for 6 min with stirring at 37°C. Mouse E2 (rE2) was expressed in *E*.*coli* (BioBasic), labeled with His-tag, isolated on a NI column, and purified on Superdex G200 to a single band by SDS-PAGE (BioBasic). Purified E2 (0–20 μg/mL), was pre-incubated with PRP/purified platelets (0.25 mL) from WTc, and WTp, E2^-/-^, and E2p^-/-^ mice for 3 min in a cylindrical glass cuvette under constant stirring.

### Plasma clotting time

The plasma recalcification time was determined by adding 20 μL of 0.25 M CaCl_2_ to 0.2 ml of citrated plasma from WTc and E2^-/-^ mice in the presence of 10 mM Tris HCl (20 μL) buffer, pH 7.4. Purified E2 (0–20μg) was pre-incubated with the plasma for 1 min at 37°C prior to recalcification.

### Clot Retraction Time

Blood was collected from the jugular vein in sodium citrate and PPP and PRP were obtained by centrifugation as described above. Samples were clotted with 1 U/mL thrombin (Sigma-Aldrich) and 5mM CaCl_2_ with gentle shaking in siliconized test tube, maintained at 37°C and photographed from 0–120min after the thrombin addition.

### Tail Bleeding/Rebleeding Assay

The bleeding/rebleeding assays ere performed as previously described [10]. The mice were anesthetized with ketamine/xylazine (90 mg/kg, 10 mg/kg), the tail prewarmed for 5 minutes in 10 mL of saline at 37°C in a water bath. The tail was lifted from the saline and a 5 mm tail segment amputated and immediately returned to the saline. Bleeding time was measured as the time between the start to the cessation of bleeding. Clot stability (rebleeding) time was measured as the time between the cessation of the bleeding and the start of the second bleeding as previously described [10].

### FeCl_3_-induced Carotid Injury Model

To induce thrombus formation in the carotid artery, a ferric chloride (FeCl_3_) model of vessel injury was employed as previously described [11]. Mice were anesthetized with ketamine/xylazine (90 mg/kg, 10 mg/kg), a midline cervical incision was made and the left common carotid artery isolated by blunt dissection. The flow probe (Transonic Systems, model 0.5PSB) was placed under the artery and when a stable baseline was reached, a 0.5 × 2 mm strip of filter paper saturated with 10% FeCl_3_ solution was applied to the surface of the carotid artery for 3 minutes. Occlusion time was determined from the addition of the FeCl_3_ solution to the occlusion of the artery (minimum blood flow). There was no difference in baseline blood flow data in carotid arteries among the mouse strains. The flow probe (Model 0.5PSB,Transonic Systems, Ithaca, NY) was in place from baseline measurements to several minutes after the stable occlusion had been reached, or stopped at 30 min if it had not occluded. Blood flow was recorded every 10 sec (Model TS420, Transonic Systems).

### Blood Cell Recovery After Bone Marrow 5-fluorouracil (5-FU) Ablation

To induce bone marrow ablation that depletes bone marrow of differentiating and maturing cells, mice were injected intravenously via the retro-orbital sinus with the cytotoxic agent, 5-fluorouracil 5-FU (250 mg/kg body weight, Pharmacia & Upjohn Company). Platelets were counted (Advia 120 Hematology Analyzer) before and 4 weeks after the injection of the 5-FU.

### Statistical Analysis

Values are expressed as the mean±SEM. Statistical differences were determined for comparison of 3 or more groups by one-way ANOVA with a Newman-Keuls post-test. For comparison of two groups, a t-test was used. *P* value of less than 0.05 was considered significant.

## Results

### Mice

**Fig 1:**
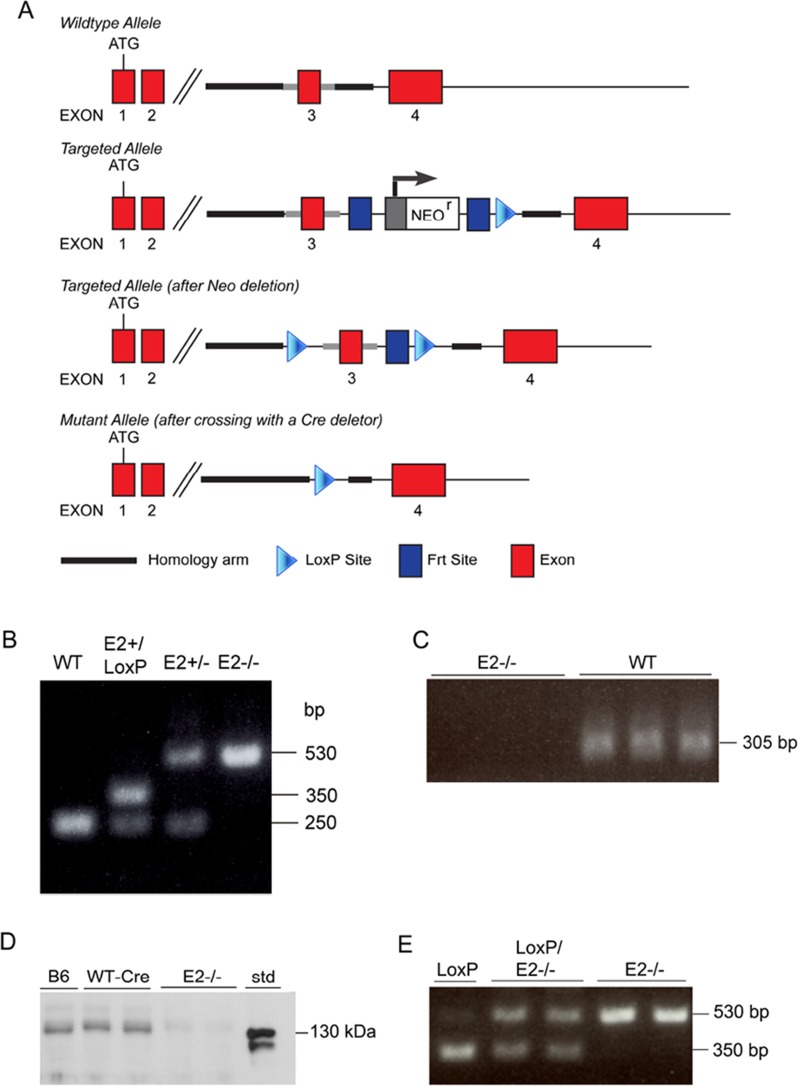
Generation of EMILIN2 Deficient Mice. A. Schematic of Wildtype Allele, Targeted Allele, Targeted Allele (after Neo deletion), Mutant Allele (after crossing with a Cre deletor). Bands: E2-/-––530bp, LoxP––350bp, WT––250bp. B. PCR EMILIN2 genotyping. CMV-cre = 100 bp (not shown) C. qRT-PCR EMILIN2 in mice. D. Western blot of 2 μL plasma from WT and E2-/-mice immunostained with primary mouse antiserum #407 (1:5000) to E2 and secondary (goat anti mouse 1:5000). Std, Standard purified E2 protein = 50ng. E. Genotyping PCR of platelet EMILIN2 conditional knockout mice. Pf4-cre = 450 bp (not shown). E2-/- (global EMILIN2 deficient), E2p-/- (EMILIN2 platelet deficient)

The homozygous E2-LoxP mice without a neocassette were crossed with the CMV-Cre to generate knockout mice (Fig. 1A). Mice were genotyped by PCR (Fig. 1B), and we confirmed by qRT-PCR that there was no E2 mRNA detected in the E2^-/-^ mice (Fig. 1C). Further, we found that there was no expression of E2 protein in the plasma of the E2^-/-^ mice by Western blot whereas a band was readily detected in plasma of ∼2.5 ng/mL, compared to the standard concentration, from WTc mice (Fig. 1D). We previously reported a low concentration of full-length E2 in platelets [14]. The homozygous E2^-/-^ mice had no overt phenotype and body weights in E2^-/-^ mice (female–18.8±0.36, male-24.3±0.40, n = 21–24) were similar to those in CMV-Cre expressing (WTc) mice (female–18.01±0.41, male–25.65±0.63,n = 19–25). There was no significant difference in platelet counts between E2^-/-^ (912±66, cell/μL, n = 5) and WTc (1000±39, cells/μL, n = 5) mice. In addition, there was no difference in the platelet recovery after bone marrow ablation between WTc (1268±61, cells/μL, n = 13) E2^-/-^ (1239±48, cells/μL, n = 9) mice. E2 conditional platelet deficient mice (E2p^-/-^) were generated by crossing the E2^*flox/flox*^ mice with Pf4-Cre recombinant mice (Fig. 1E). The E1 deficient mice have been previously described [17,18,19] and displayed no overt phenotype.

### Platelet Aggregation is Impaired in E2^-/-^ and E2p^-/-^ Mice

**Fig 2:**
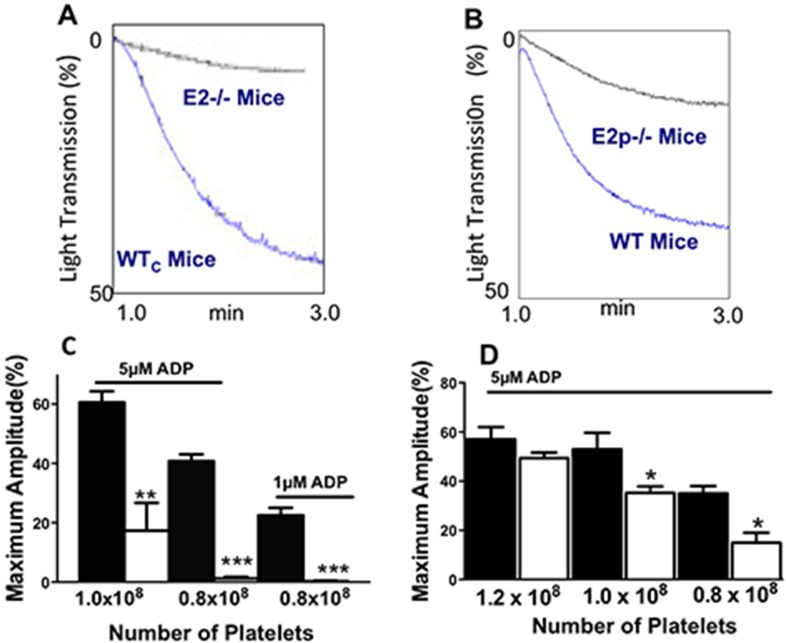
Aggregation of platelets from E2^-/-^ and E2p^-/-^ mice. A,C. E2^-/-^ mice. B,D. E2p^-/-^ mice. A,B. Representative (3–5 experiments repeated 2–3 times with each platelet preparation) aggregometer tracing in PRP induced by 5μM ADP with platelets adjusted to 1.2x10^8^ per ml. C,D. Maximum amplitude from the platelet aggregometry tracings. The aggregation of PRP was induced with 1 μM or 5μM ADP added to PRP with platelets at adjusted to 0.8–1.2x10^8^ per mL pooled from 3 mice. Bars are mean±SEM, performed in triplicate. Statistical analysis, one-way ANOVA, Newman-Keuls post-test **P = 0.01, ***P = 0.001. E2^-/-^ (EMILIN2 deficient), E2p^-/-^ (EMILIN2 platelet deficient)

Previously, we reported that platelet aggregation is inhibited with an antibody to E2 [14]. Platelet aggregation was measured in the E2^-/-^ and E2p^-/-^ mice. Platelet concentration in PRP was adjusted with PPP from mice of the same genotype. With 2x10^8^ platelets in the PRP and 5μM ADP, no difference in the aggregation of E2^-/-^ and WTc mice was detected (not shown). However, at lower platelet (0.8x10^8^) trations, platelet aggregation was negligible in the E2^-/-^ mice while still robust with WTc controls (Fig. 2A,B). Platelet aggregation in E2p^-/-^ mice displayed a similar defect; reduced aggregation in response to ADP at lower (1.0 or 0.8x10^8^ platelets/mL) but not higher platelet concentrations (Fig. 2C,D). However, there was a smaller difference between WTp and E2p^-/-^ platelet aggregation than observed in the E2^-/-^ mice, suggesting both platelets and plasma may contribute to the maximal effect of E2 deficiency on the aggregation response.

**Fig 3:**
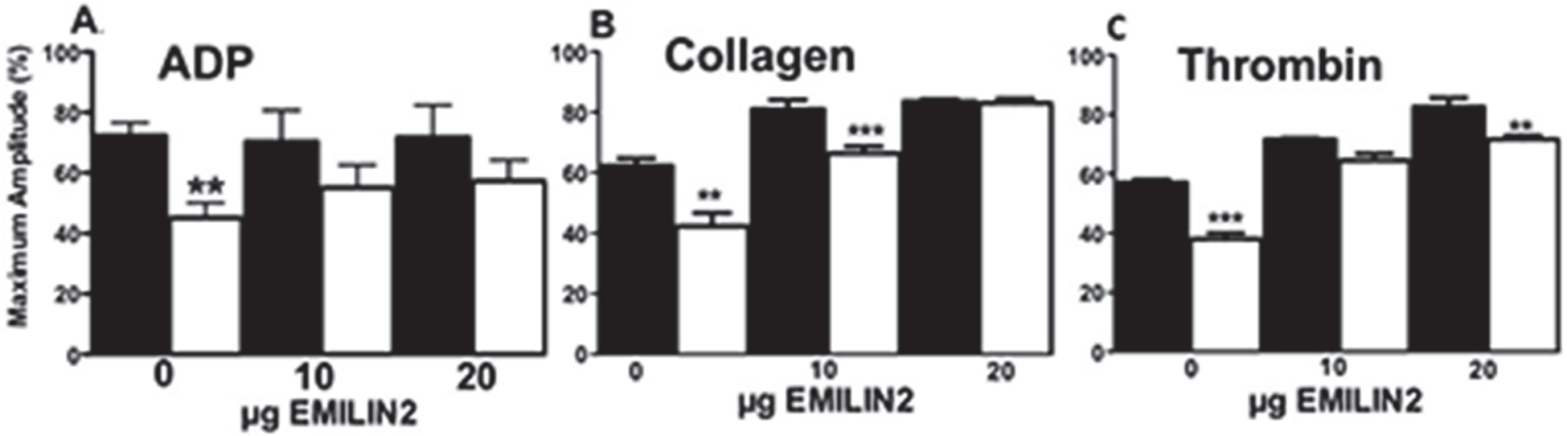
Platelet aggregation is impaired in E2^-/-^ mice in response to ADP, collagen or thrombin and E2 increases aggregation of both WTc and E2^-/-^ platelets. A,B,C. Platelet aggregation measured in platelet rich plasma (1 x 10^8^ platelets/assay). Maximum amplitude (% Light Transmission). A. 2.5 μM ADP, B. 1 μg collagen, C. 0.5 U/mL thrombin. Bars are the mean±SEM of 3–5 separate experiments performed in triplicate of platelets pooled from 3 mice. Statistical analysis, one-way ANOVA. **P < 0.001 ***P < 0.001. WTc (black bar), E2^-/-^ (white bar).

Suppressed aggregation of E2^-/-^ platelets was observed in response ADP (Fig. 3A), to low doses of collagen to platelets in PRP (Fig. 3B) and with washed platelets in response to thrombin (Fig. 3C), suggesting that the effects of E2 were not restricted to a single agonist. Adding purified rE2 to platelets increased platelet aggregation in WTc and E2^-/-^ mice in response to ADP, collagen, and thrombin. While rE2 increased platelet aggregation, the effect was not only observed in the absence of E2; rE2 also tended to increase the aggregation of WTc platelets in response to collagen and thrombin (Fig. 3B,C). These results suggest that platelet and plasma E2 can influence platelet aggregation. However, E2 alone had no effect on platelet aggregation at concentrations as high as 20μg (not shown).

### Platelet Aggregation is Impaired by E2^-/-^ Plasma

**Fig 4:**
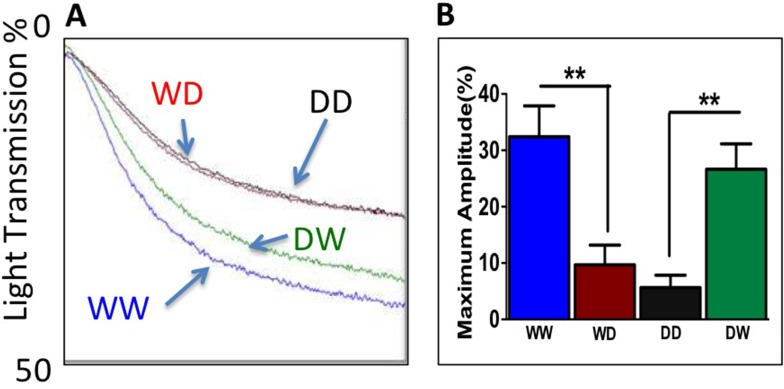
Platelet and Plasma Mixing Experiments. A,B. WW–WTc platelets and plasma, WD–WTc platelets and E2^-/-^ plasma, DD–E2^-/-^ platelets and plasma, DW–E2^-/-^ platelets and WTc plasma. Number of platelets per assay, 1x10^8^ per mL, pooled from 3 mice, 1μM ADP. Bars are mean±SEM, n = 3 performed in triplicate. Statistical analysis, one-way ANOVA, Newman-Keuls post-test, **P = 0.01

To determine relative contribution of platelet and plasma E2 to the impaired aggregation in the E2^-/-^ mice, E2^-/-^ platelets and/or plasma were replaced with the platelets (PRP) and plasma (PPP) from the WTc mice (Fig. 4A,B). Adding WTc platelets to E2^-/-^ plasma reduced the maximum amplitude of platelet aggregation (WD, red curve, red bar) compared to the aggregation response obtained with both WTc platelets and WTc plasma (WW, blue curve and blue bar), and the reduced aggregation was similar to the E2^-/-^ platelets and plasma (DD, black curve, black bar). When the platelets from the E2^-/-^ mice were added to the WTc plasma (DW, green curve, green bar), aggregation was restored to homologous WTc level. These data suggest that plasma E2 itself or an E2-dependent factor in plasma is primary pathway by which E2 suppresses platelet aggregation.

### E2 Inhibits Clotting Time of Mouse and Human Platelet-rich Plasma

**Fig 5:**
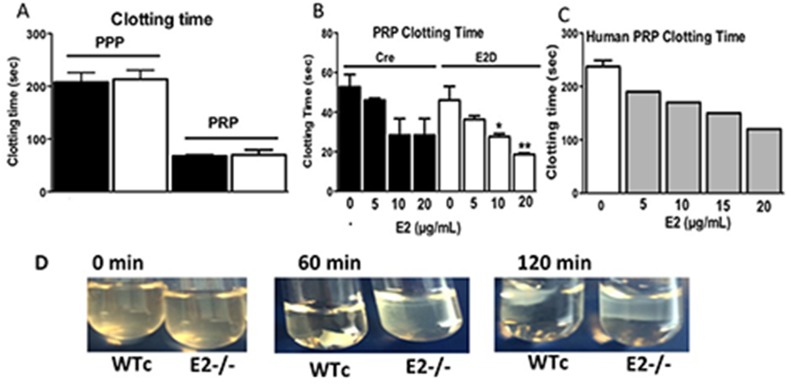
Role of E2 in Clotting Time and Clot Retraction. A. Clotting time in WTc and E2^-/-^ mouse PPP and PRP was induced by CaCl_2_. B. Clotting time of PRP upon addition of varying concentrations of recombinant EMILIN2 (rE2). rE2 protein was preincubated with PRP or PPP prior to addition of CaCl_2_. C. Clot retraction. Plasma clotted with 1 U/mL thrombin and 5mM CaCl_2_, maintained at 37°C, and photographed from 0–120 min after the thrombin addition.

In view of the association of the QTL on human Chr 18 where E2 resides with thrombosis, we tested other ways in which E2 or its deficiency might influence thrombus formation. We measured clotting time in the E2^-/-^ mice in both PPP and PRP. The clotting time was similar in the WTc and E2^-/-^ mice (Fig. 5A). Addition of E2 decreased clotting time of platelet-rich plasma from WTc and E2^-/-^ mice (Fig. 5B). This effect was more pronounce in the E2^-/-^ PRP as the clotting time was significantly shorter with 10 and 20μg/mL added E2. In human PRP, added E2 led to a dose dependent decrease in clotting time. At 20μg/mL added E2, clotting time was reduced 2-fold (Fig. 5C). Thus, addition of the rE2 to the clotting assay is similar to the effect of E2p^-/-^ PRP (with E2) on platelet aggregation where the aggregation is less severe than for E2^-/-^ PRP (Fig. 2D).

We also measured the retraction of clots formed in PRP from WTc and E2^-/-^ mice. The photographs shown in Fig. 5D show markedly delayed retraction of E2^-/-^ clots compared to the WTc clots at 60 min (Fig. 5D). Clot retraction is a consequence of integrin outside-in signaling is affected by E2 deficiency [20].

### Thrombosis

**Fig 6:**
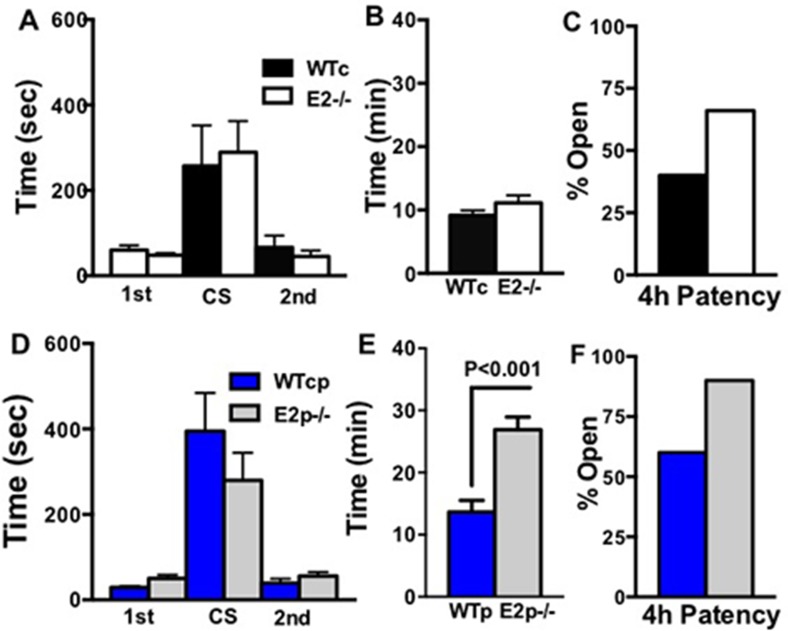
Bleeding and Thrombus Formation in E2^-/-^ and E2p^-/-^ Mice. A. D. Tail Bleeding/Rebleeding Assay. Bleeding time (1^st^) is between the start of the bleeding and cessation of the bleeding. Clot stability (rebleeding) time (CS) is measured as the time between the cessation of the bleeding and the start of the second bleeding time (2^nd^). Bars are mean±SEM, n = 9–11. Statistical Analysis, One-way ANOVA. B, E. Carotid Occlusion Time. Bars are mean±SEM, n = 9–10. Statistical analysis, t-test. C,F. Patency (percent mice with open carotid 4hr after treatment), WTc mice (4/10), E2^-/-^ mice (6/9). WTp mice (3/5), E2p^-/-^ (9/10).

To determine if the E2^-/-^ and the E2p^-/-^ mice have a bleeding or thrombosis disorder, we performed tail bleeding/rebleeding assay [10] on these animals. Tail bleeding times, clot stability times, or rebleeding times were not significantly different assay (Fig. 6A). Occlusion time after FeCl_3_ treatment [21] (Fig. 6B) also were not significantly different in the E2^-/-^ mice and WTc mice. However, at 4 hrs after FeCl_3_ carotid injury, patency in the E2^-/-^ mice was higher than in WTc mice (Fig. 6C). There was no difference in bleeding, clot stability or rebleeding in E2p^-/-^ compared WTp mice (Fig. 6D). In the E2p^-/-^ mice, unlike the E2^-/-^ mice, we found occlusion time after FeCl_3_ carotid injury was nearly 3-fold longer in the E2p^-/-^ mice compared to WTp mice (Fig. 6E). An increased occlusion time suggested that E2p^-/-^ mice had impaired thrombus formation and E2 in the plasma inhibited thrombus formation. The number of mice with open carotids (patency) 4 hr after occlusion was elevated in both of the E2^-/-^ and E2p^-/-^ mice (Fig. 6F) compared to the WTp mice, suggesting that thrombi were unstable in the both E2^-/-^ and E2p^-/-^ mice.

### E1 Deficiency Increases Clotting Time and Speeds Clot Retraction

**Fig 7:**
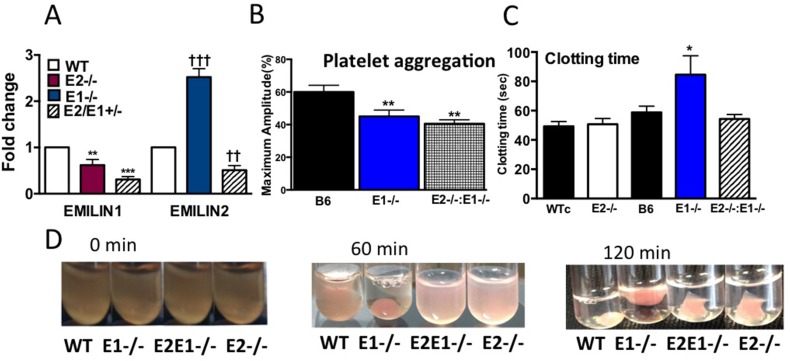
Quantitative RT-RCR, Clotting Time, and Clot Retraction in E2^-/-^, E1^-/-^ and E2^-/-^:E1^-/-^ mice. A. Quantitative RT-RCR relative to GAPDH, n = 3. B. EMILIN1 in E2^-/-^ mice. **P 0.01, ***P <0.001, E2 in E1^-/-^ mice. ††P 0.01,†††P <0.001. B. Platelet aggregation. 2.5 μM ADP, 1 x10^8^ platelets. Maximum Amplitude (% Light Transmission). 3–5 experiments repeated 2–3 times per experiment. C. Clotting time. n = 3. **A-C.** Bars are mean±SEM. One-way ANOVA, Newman-Keuls post-test, *P = 0.05, **P = 0.01. C. Clot retraction. Plasma clotted with 1 U/mL thrombin and 5mM CaCl_2_, maintained at 37°C, and photographed from 0–120 min after the thrombin addition. Representative photographs of 3 experiments.

E1 has high homology to E2 [21] and dimerizes with E2 [1,15]. We generated E2^-/-^:E1^-/-^ double knockout mice and evaluated platelet aggregation, clotting time and clot retraction in E1 and E2^-/-^:E1^-/-^ mice. Expression of E2 and E1 mRNA were quantified by qRT-PCR in WT (B6), E2^-/-^ and E1^-/-^ and double heterozygous E2^-/-^:E1^-/-^ mice (Fig. 7A). E1 expression in E2^-/-^ and E2/E1/H was significantly lower than WT mice. However expression of E2 in E1^-/-^ mice was nearly 3-fold higher than WT mice. In E1^-/-^ and E2^-/-^:E1^-/-^ mice platelet aggregation was suppressed in ADP PRP (Fig. 7B) similar suppressed aggregation in E2^-/-^ mice. Platelet aggregation was also suppressed in E1D and E2^-/-^:E1^-/-^ mice with collagen stimulated platelets in PRP and with washed platelets stimulated with thrombin (not shown) similar to the E2^-/-^ suppression of platelet aggregation (Fig. 3). ADP platelet stimulated aggregation in the E2^-/-^:E1^-/-^ mice was also suppressed. These results suggest that both E1 and E2 influence platelet aggregation.

Unlike clotting time in E2^-/-^ compared to WTc mice, clotting time in E1^-/-^ mice was markedly increased compared to its control B6 mice. In contrast, E2^-/-^:E1^-/-^ mice were similar to the E2^-/-^ mice, showing no difference in clotting time compared to B6 mice or WTc mice. E2^-/-^ clotting time in PRP and PPP was similar to the WTc, but clotting time was increased in both PRP and PPP in the E1^-/-^ mice compared to B6 mice (Fig. 7B and S1 Fig.). Clot retraction occurred more quickly in the E1^-/-^ mice than in control mice, suggesting E1 inhibits clot retraction, but clot retraction in E2^-/-^:E1^-/-^ was delayed similar to the delayed clot retraction in the E2^-/-^ mice. These results suggest that the affect of E2 and E1 on clotting time and clot retraction is different and that their interaction may determine their overall regulation of these responses.

## Discussion

Previously, E2 was identified in mouse and human QTLs as a modifier of thrombosis[14] and that inhibition of E2 impaired platelet aggregation. The findings of this study in E2 deficient mice indicate that: 1) E2 deficiency suppresses platelet aggregation induced by ADP, collagen, and thrombin; 2) both E2 deficient platelets and plasma contribute to this suppression of platelet aggregation; 3) purified E2 preferentially decreased E2^-/-^ clotting time; 4) in an *in vivo* thrombus model mice with deficient E2 platelets only have carotid occlusion times 3-fold longer than their wild-type control mice and thrombus stability was reduced in both E2^-/-^ and E2p^-/-^ mice; 5) expression of E2 is 3-fold higher in E1^-/-^ mice than in B6 mice; and 6) while E2^-/-^ and E1^-/-^ platelets had suppressed platelet aggregation compared to their WT control platelets, they have opposing effects on clotting time and clot retraction.

The EMILIN proteins are a family of extracellular matrix glycoproteins [1] that includes E1, and MMNR1, MMN2, and E2, that all share three common domains: C-terminal gC1q domain, coiled-coil domain, and N-terminal cysteine-rich domain (EMI domain). Many of the ECM elastin/microfibril associated proteins [22], such as fibulin 1 and MAGP1, are multifunctional and play roles not only in elastogenesis and vascular architecture, but also in hemostasis and thrombosis [7,23]. MNR1 levels increase 7-fold on outer surface of the platelets with platelet activation [8] and binds Factor V in platelet α-granules. PEAR1, which contains an N-terminal EMI domain, is a platelet membrane protein and has been shown to stabilize sustain platelet aggregates [24]. A role of E2 as a platelet associated protein is consistent with the multifunction roles of other microfibril-associated proteins and its highly interreactive Clq and EMI domains. In the present study, we detected E2 in plasma by Western blot and previously detected a low concentration of full-length E2 protein in platelets [14]. Rowley et al. [25] reported the E2 mRNA expression in platelets, but 2-fold lower than E1 and 20-fold lower than MMNR1. E2 protein was reported to present in platelets and the concentration in plasma was 4-fold higher [26]. Our findings would support a functional role for E2 in plasma [26].

Our results indicate that E2^-/-^ mice have reduced platelet aggregation in response to ADP, collagen and thrombin, and purified E2 added to platelets restored the impaired aggregation of E2^-/-^ platelets. While maximum amplitude of platelet aggregation with ADP was less in WTc mice at reduced platelet number and lower ADP, the suppressed aggregation in the E2^-/-^ and E2p^-/-^ mice was platelet concentration dependent. Other studies have demonstrated platelet count dependent aggregation [27,28] and it is not unusual that platelet aggregation defects become evident at low concentrations of platelets and/or agonists [29,30,31,32]. The decreased aggregation only at dilute platelet concentrations in the E2^-/-^ mice, the less severe suppression of platelet aggregation in the E2p^-/-^ mice, as well as the increasing platelet aggregation upon addition of E2 to plasma suggests that there is a dampener of platelet aggregation in plasma and E2 may help to neutralize this inhibitor. Clotting time was not different in plasma or PRP in E2^-/-^ mice, suggesting that coagulation is not impaired in the E2^-/-^ mice. If the E2 deficiency only altered platelet aggregation, then aggregation would be equivalent in the E2^-/-^ and E2p^-/-^ platelets and E2^-/-^ plasma would not inhibit platelet aggregation of the WTc platelets. The markedly delayed clot retraction in the E2^-/-^ mice supports the notion that E2 attenuates an inhibitor of platelet responses in plasma. Work is underway to identify this hypothetical suppressor of platelet aggregation assuming that it is a binding partner of E2.

E1 is highly homologous to E2, with 70% homology in their EMI and 75% in their Clq domains [21]. E2 was identified as a binding partner of E1 and it was suggested that they might interact to form a heterotrimer and co-localize in the ECM [15,33]. Both E1 and E2 contain a binding motif for integrin**α**4**β**1 [34,35], which is not present on platelets. Like E2 deficiency, E1 deficiency also suppressed platelet aggregation. However, E2 and E1 have opposing effects on clot retraction with E2 increasing and E1 decreasing clot retraction. Double deficient (E2^-/-^:E1^-/-^) mice had suppressed platelet aggregation suggesting that both E2 and E1 contribute to platelet aggregation. The E2^-/-^:E1^-/-^ plasma retracted clots slowly like the E2^-/-^ plasma in contrast to the rapid retraction with the E1 system, suggesting that E2 dominates this effect.

Previously, we found that an antibody to E2 decreased platelet aggregation in B6 mice.

Our results with suppressed aggregation in the E2^-/-^ mice are consistent with this initial observation. In the previous study we also reported that the antibody was localized in the thrombus and here we report decreased thrombus stability in the deficient mice. Patency of the carotid after thrombosis occurred in a higher percent of deficient mice than control mice. In a preliminary study we reported cardiac abnormalities, such as pulmonary regurgitation or ventricular septal defects in E2, E1 single deficient mice and double deficient mice unpublished (Hoover-Plow unpublished, 2014). In humans, pulmonary regurgitation or ventricular septal defects may be a precursor to the development of heart failure [36]. Elevated platelet activation and hypercoagulation are common in HF and contribute to the thromboembolism susceptibility [37,38,39], such as PACE, (a syndrome associated with large hemangioma). The role of E2 in heart failure and hypercoagulation is not known.

**Limitations of study and future directions.** This study of E2 deficient mice has identified an important role for E2 in the regulation of platelet aggregation and clot retraction. However, further studies are needed to define how E2 regulates platelet aggregation and how E2 and E1 cooperate to influence thrombosis and homeostasis.

## Supporting Information

S1 Fig Bleeding and Thrombus Formation in E1-/- Mice. **A**. Tail Bleeding/Rebleeding Assay. 1^st^-first bleeding, CS-Clot stability, 2^nd^-second bleeding, n = 23–29. B. Carotid Occlusion Time. Bars are mean±SEM, n = 16–25. Statistical analysis, t-test. C. Patency (percent mice with open carotid 4hr after treatment), B6-mice (15/25), E2D mice (14/15).
(TIF)

S1 File Fig. S1 Methods. Tail Bleeding/Rebleeding Assay.
(DOCX)
