# Supplementary figures and images for: EMILIN2 Regulates Platelet Activation, Thrombus Formation, and Clot Retraction
Source: PLoS One. 2015 Feb 6;10(2):e0115284. doi: 10.1371/journal.pone.0115284 (PMC4319747; doi:10.1371/journal.pone.0115284)

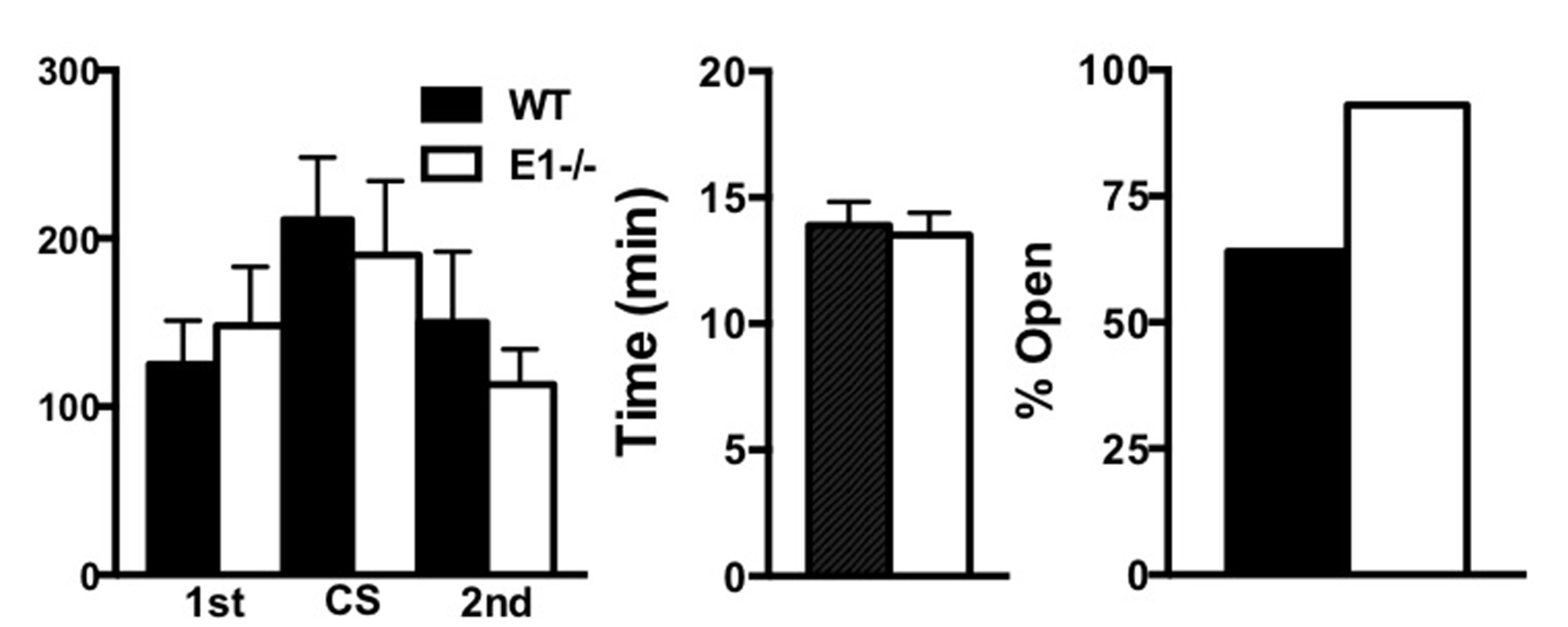

Supplement: S1 Fig — A. Tail Bleeding/Rebleeding Assay. 1st-first bleeding, CS-Clot stability, 2nd-second bleeding, n = 23–29. B. Carotid Occlusion Time. Bars are mean±SEM, n = 16–25. Statistical analysis, t-test. C. Patency (percent mice with open carotid 4hr after treatment), B6-mice (15/25), E2D mice (14/15). (TIF) [file pone.0115284.s001.tif]
