# Supplementary material for: EMILIN2 Regulates Platelet Activation, Thrombus Formation, and Clot Retraction
Source: PLoS One. 2015 Feb 6;10(2):e0115284. doi: 10.1371/journal.pone.0115284 (PMC4319747; doi:10.1371/journal.pone.0115284)
Supplement: S1 File — Tail Bleeding/Rebleeding Assay. (DOCX) [file pone.0115284.s002.docx]

**Supporting Information File**

**Methods**

**Tail Bleeding/Rebleeding Assay**

The bleeding/rebleeding assay was performed as previously described [Hoover-Plow J, Shchurin A, Hart E, Sha J, Hill AE, et al. (2006) Genetic background determines response to hemostasis and thrombosis. BMC Blood Disord 6: 6]. The mice were anesthetized with ketamine/xylazine (90 mg/kg, 10 mg/kg), the tail prewarmed for 5 minutes in 10 mL of saline at 37°C in a water bath. The tail was lifted from the saline and a 5 mm tail segment amputated and immediately returned to the saline. Bleeding time was measured as the time between the start of the bleeding and cessation of the bleeding. Clot stability (rebleeding) time was measured as the time between the cessation of the bleeding and the start of the second bleeding.
